# Infectious Disease and Primary Care Research—What English General Practitioners Say They Need

**DOI:** 10.3390/antibiotics9050265

**Published:** 2020-05-20

**Authors:** Donna M. Lecky, Steve Granier, Rosalie Allison, Neville Q. Verlander, Simon M. Collin, Cliodna A. M. McNulty

**Affiliations:** 1 Primary Care and Interventions Unit, Public Health England, Gloucester GL1 1DQ, UK; rosie.allison@phe.gov.uk (R.A.); cliodna.mcnulty@phe.gov.uk (C.A.M.M.); 2 Whiteladies Medical Group, Bristol BS8 2PU, UK; steve.granier@nhs.net; 3 Statistics, Modelling and Economics Department, Public Health England, London NW9 5EQ, UK; neville.verlander@phe.gov.uk; 4 HCAI & AMR Division, Public Health England, London NW9 5EQ, UK; simon.collin@phe.gov.uk

**Keywords:** primary care, antibiotic resistance, antibiotics, prescribing guidance, survey

## Abstract

Background: Infections are one of the most common reasons for patients attending primary care. Antimicrobial resistance (AMR) is perhaps one of the biggest threats to modern medicine; data show that 81% of antibiotics in the UK are prescribed in primary care. Aim: To identify where the perceived gaps in knowledge, skills, guidance and research around infections and antibiotic use lie from the general practitioner (GP) viewpoint. Design and Setting: An online questionnaire survey. Method: The survey, based on questions asked of Royal College of General Practitioners (RCGP) members in 1999, and covering letter were electronically sent to GPs between May and August 2017 via various primary care dissemination routes. Results: Four hundred and twenty-eight GPs responded. Suspected Infection in the elderly, recurrent urinary tract infection (UTI), surveillance of AMR in the community, leg ulcers, persistent cough and cellulitis all fell into the top six conditions ranked in order of importance that require further research, evidence and guidance. Acute sore throat, otitis media and sinusitis were of lower importance than in 1999. Conclusion: This survey will help the NHS, the UK National Institute for Health and Care Excellence (NICE) and researchers to prioritise for the development of guidance and research for chronic conditions highlighted for which there is little evidence base for diagnostic and management guidelines in primary care. In contrast, 20 years of investment into research, guidance and resources for acute respiratory infections have successfully reduced these as priority areas for GPs.

## 1. Introduction

Activity in general practice has increased significantly over the past five years, with the average person visiting their general practice around six times a year [1]. Most primary care consultations (52%) are conducted by a general practitioner (GP) and 82% of all appointments are face to face [2]. However, overall patient satisfaction with general practice has declined [1]. Initiatives to improve patient care mean that multiple actions are needed for each patient, e.g., screening, monitoring and other disease management tasks, which presents a challenge to GPs working to a 10-min consultations model.

Antimicrobial resistance (AMR) is perhaps the biggest threat to modern medicine and it will still be even after the COVID-19 pandemic. The majority of antibiotics in England, 81%, are prescribed in primary care [3] and is likely to increase during the COVID-19 pandemic due to remotely prescribed and broad-spectrum antibiotics. Across the UK, general practice is the first port of call for many people presenting with an infectious disease [3,4,5]; penicillins are the most commonly prescribed antibiotic group in this setting (46.5%) by items prescribed per 1000 inhabitants per day, followed by tetracyclines (13%), then macrolides (11.7%) [3]. Similar trends have been observed in other UK nations [4,5].

The overprescribing of antibiotics is a major driver for antibiotic resistance. In 2018, research estimated that at least 20% of antibiotics are prescribed inappropriately in England, many of these for respiratory tract infections (RTIs) [6]. It was found that 41% of acute cough consultations were prescribed antibiotics when only 10% were deemed appropriate [7]. A recent systematic review highlighted diagnostic uncertainty as a contributing factor for overprescribing for acute RTIs [8]. Researchers also found that consistently available national guidelines on antibiotic prescribing, were regarded as important by clinicians for their prescribing decision making. As such, this study aims to identify current gaps in knowledge, skills, guidance and research from the GP’s point of view as [1,2,6,7] this will facilitate improved antimicrobial stewardship.

In a 1999 survey of GPs, we found that genital chlamydia infection, antibiotic resistance surveillance, vaginal discharge, leg ulcers, sinusitis, otitis media/externa, dyspepsia/Helicobacter pylori, Creutzfeldt–Jakob disease (CJD) and tonsillitis were the top 10 priorities for improvements to diagnostic tests, and stronger evidence on which to base treatment decisions [9,10]. The present study will also compare findings from the 1999 study.

## 2. Results

### 2.1. Response Rate

Of those who opened the online survey, 12% (428/3526) completed all questions, not all participants completed all questions.

**Figure 1:**
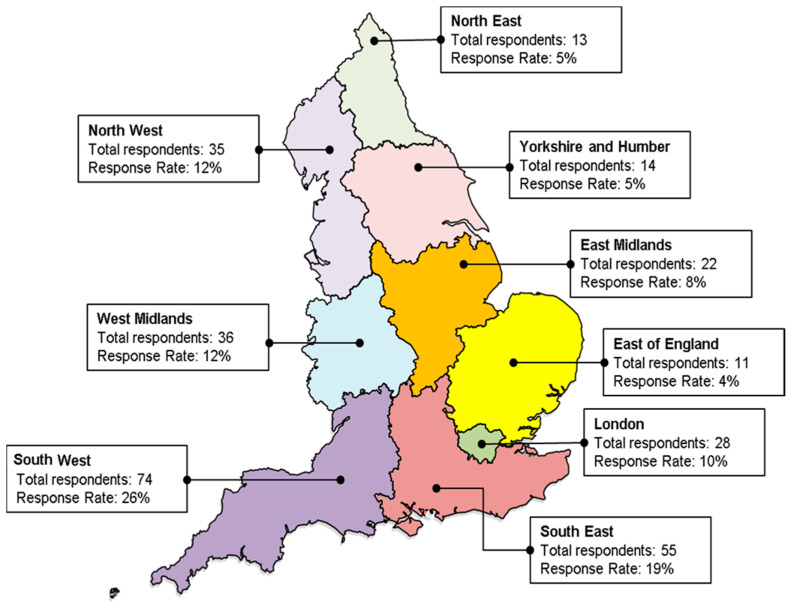
Survey response rate by area in England.

For those who completed the demographic data section of the survey, 97% (349/361) of respondents were from England, with 1% from each of the other devolved administrations (Scotland 5/361; Wales 4/361; Northern Ireland 3/361). The response rate by region of England is shown in Figure 1. 15% (54/360) of respondents self-identified as being from rural practices.

In total, 48% (174/361) of respondents stated that they were from a research practice. A total of 32% (118/370) reported receiving the survey from the Royal College of General Practitioners (RCGP), 22% (80/370) from the CRN, 31% (115/370) from their local CCG, 7% (25/370) from a colleague, 3% from RCGP First 5 group (10/370) and 6% (22/370) from another source. Gender, age and years in practice data can be found in Table 1.

### 2.2. Representativeness of the Data

Table 1 demonstrates that there were no differences in the characteristics of GPs in the final sample of survey respondents and those of all GPs in the sample frame, in terms of age and sex. There was a slight over-representation of GPs stating they were from a research practice compared with GPs in the sample frame and respondents were overrepresented from the South West and underrepresented from the East of England; Yorkshire and Humber; and London.

### 2.3. Condition Ranking

**Figure 2:**
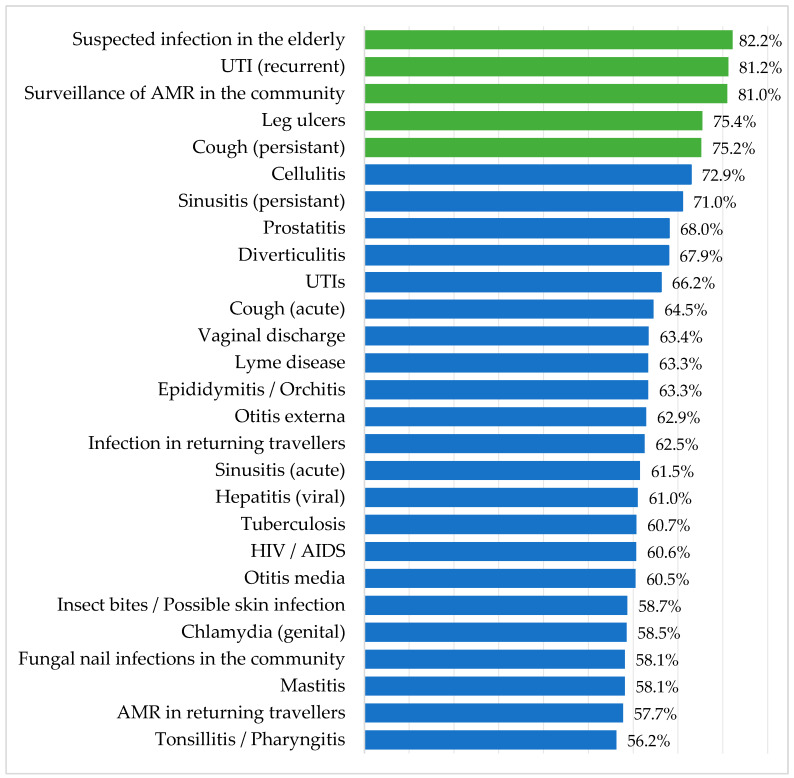
Condition ranking presented as a percentage of the total possible score for each condition. Condition ranking is the total scores for each illness (where 1 = further research unimportant, 5 = further research very important) were converted into a percentage by dividing the total score by the maximum possible score, i.e., as if all respondents indicated that further research into that illness was ‘very important’.

Of the 27 named conditions/illnesses, suspected infection in the elderly (82.2%), recurrent urinary tract infection (UTI) (81.2%), surveillance of antibiotic resistance in the community (81.0%), leg ulcers (75.4%), and persistent cough (75.2%) were the five most highly rated illness/conditions where respondents felt they required more evidence to support their daily practice. Weighted scores for all 27 named conditions are shown in Figure 2. Ranking did not differ between research and non-research practices (Table A1). Condition ranking compared to the 1999 study can be seen in Table 2.

### 2.4. Top Three Illnesses/Conditions That Require Further Research, Evidence and Guidance

From the list of 21 named conditions, respondents were asked to identify the top three illnesses/conditions they felt required further research, evidence and guidance. Table 2 illustrates that the five most frequently named illnesses/conditions by GPs that require further research, evidence and guidance, were similar to the top five ranked illnesses/conditions. A total of 115 respondents added both suspected infection in the elderly and surveillance of antibiotic resistance in the community in their top three conditions, resulting in these conditions being ranked joint first place, followed by recurrent UTI, persistent cough, cellulitis and leg ulcers, in that order. Ranking did not differ between research and non-research practices (Table A1).

#### Other Conditions

Participants (55%; 239/234) identified over 170 ‘other’ areas that required further research. The most frequently identified areas were mental health (13.07%; 63 mentions), pain management (6.93%; 35 mentions), skin conditions (5.88%; 30 mentions) and chronic fatigue or fibromyalgia (5.54%; 28 mentions).

### 2.5. Type of Evidence, Research and Guidance Needed

Table 3 shows rankings for which areas of research (near patient antibiotic resistance test; clinical scores to help inform management; or point of care prognostic tests), evidence (evidence base for antibiotic treatment; evidence base for self-care and non-antibiotic treatment) or guidance (improved treatment guidelines) respondents felt were required for each condition/illness. The top three priorities across all 27 named conditions were the ‘need for better evidence base for antibiotic treatment’ (exceptions: viral hepatitis and HIV/AIDS); the ‘need for improved treatment guidelines for primary care staff’ (exceptions: acute cough and surveillance of AMR in the community); and the ‘need for better evidence base for self-care and non-antibiotic treatment in primary care’ (exceptions: genital chlamydia, Lyme disease and suspected infection in the elderly and tuberculosis (TB)). There was little variation between the ranking of the ‘need for better clinical scores to help inform management in primary care’ (exceptions: viral hepatitis, otitis externa, prostatitis, tonsillitis and TB) and the ‘need for better point of care prognostic tests in primary care’ (exception: genital chlamydia), with both being ranked in either the 4th or 5th position. The need for better near patient antibiotic resistance test in primary care was the lowest ranked respondent priority across all conditions/illnesses (exceptions: AMR in returning travelers and genital chlamydia).

## 3. Discussion

The conditions for which a GP said they wanted more evidence to support their daily practice and require further research, evidence and guidance were: suspected infection in the elderly; recurrent UTI; surveillance of antimicrobial resistance in the community; leg ulcers; persistent cough; and cellulitis. The need for a better evidence base for antibiotic treatment in primary care; the need for improved treatment guidelines for primary care staff; and the need for better evidence base for self-care and non-antibiotic treatment in primary care were considered the most important service developments. The need for better point-of-care prognostic test, clinical scores to inform management, and near patient antibiotic susceptibility tests were considered less important.

### 3.1. Strengths and Limitations

To improve response rates, the survey was disseminated via relevant GP channels in England but we have no information as to how many GPs actually received the invitation to participate in the survey. Survey site data suggest that internal surveys generally receive a 30–40% response rate compared with 10–15% for external surveys [12,13], which is in line with our findings regarding how many people opened the survey vs. how many people actually completed it.

Demographic data indicates that respondents were generally representative of GPs in England by age, gender and years in practice distribution. Percentage response rates for age and gender were similar to national data [11,14], and our findings show no differences between GP practice research status and rating of importance of more evidence.

We did not collect data on respondent workplace and have assumed independence in all analysis which may be considered a limitation.

The provision of a named list of conditions helped reduce seasonal or respondent bias towards specific conditions. The ‘other’ option allowed respondents to add conditions they felt were important but were not on the main list.

### 3.2. Comparison with Existing Literature

The last UK GP survey of this nature was conducted in 1999 and found genital chlamydia infection to be the number one priority for ‘improvements to diagnostic tests, evidence on which to base treatment, and guidance’ [9,10]. Interestingly a 40-fold variation in testing rates across GP practices was observed at this time [15]. The drop to position 23 for Chlamydia, and the drop from the 4th position to 12th position for vaginal discharge in the latest survey, may be attributed to the introduction of evidence-based national guidelines and standards for UK specialists in genitourinary medicine [16] and STIs [17] and the establishment of the national chlamydia screening programme (NCSP) in 2002 [18]; the latter of which contributed to a reduction in the prevalence and average duration of infections following implementation [19].

Other conditions that have dropped out of the top 10 position since the 1999 survey included a range of respiratory tract infections (RTIs), suggesting that GPs feel the evidence base for diagnosis and treatment of these conditions is adequate. Much research has gone into developing evidence-based guidelines for RTIs [20,21] and clinical prediction tools in recent years for self-limiting RTIs [22,23,24,25,26]. Health professional training workshops and toolkits [27] may also account for the increase in GP confidence to treat these infections. Public education campaigns aimed at reducing patient expectations for antibiotics and focussing on RTIs have resulted in a decrease in the expectation of antibiotics for these conditions and for consultations with a cough or cold [28].

Venous leg ulcers and persistent sinusitis have remained in the top 10 with the need for improved treatment guidelines a named priority area. A recent systematic review [29] found only four clinical practice guidelines worldwide (none in England) on venous leg ulcers, between 1999 and 2016, considered to be of adequate quality for clinical use. There have been few clinical trials on the antibiotic treatment of leg ulcers; more research has gone into non-antibiotic treatment and the chronic relapsing nature of the condition, highlighting the complexity of treatments for GP staff to follow [30]. In February 2020, the UK National Institute for Health and Care Excellence (NICE) developed antibiotic prescribing guidance for leg ulcer infection [31].

For persistent sinusitis, GPs ranked the need for a better evidence base for self-care and non-antibiotic treatment as a priority area. Design variation in studies investigating the effects of antibiotic use for chronic rhinosinusitis make drawing firm conclusions in systematic reviews difficult [32,33,34]. A Cochrane review concluded that there was little evidence that systemic antibiotics are effective in patients with chronic rhinosinusitis and that more research in the field is required [33]. NICE published specific Managing Common Infection guidance for acute sinusitis [35] of less than four weeks with sudden onset of symptoms, but there is no UK guidance available for persistent sinusitis.

An observed increase in the incidence of blood stream infections associated with urinary tract infections (UTI) and increasing AMR may account for the elevation of recurrent UTI to 2nd place in 2017 from 18th in 1999 [9]. During the time of the survey, NHS England implemented a mandate to reduce inappropriate antibiotic prescribing for UTI in primary care [36], which may account for the greater interest in the treatment of UTIs, which represent 1–3% of UK primary care consultations. GPs in our survey recorded a ‘need for a better evidence base for self-care and non-antibiotic treatment’ and ‘the need for a better evidence base for antibiotic treatment’ as their two priority areas, followed by ‘the need for improved treatment guidelines’. Although antibiotic prescribing guidelines have been available for suspected bacterial UTIs in the UK, none focussed on recurrent UTIs. Since this study was conducted, NICE have published antimicrobial prescribing guidance for lower, upper, recurrent and catheter-associated UTI [37].

Interestingly, GPs’ ranking of Tuberculosis (TB), 19/27 (*n* = 411), remains unchanged, with only eight individuals placing it in their top three most important conditions. This is surprising as, due to its resistance to a wide range of antimicrobials, TB is named in the Department of Health 5-year strategy [38] and 20-year vision [39] for antimicrobial resistance. The lower priority of TB in this survey may be because TB infections are mostly diagnosed in the London area [40], and only 10% of our respondents were from this region.

Suspected infection in the elderly, prostatitis, and diverticulitis were ranked in the top 10 of conditions for which GPs required evidence to support their daily practice; (ranked first, eighth and ninth respectively). These conditions were not given as an option in the 1999 study therefore we cannot compare our findings. For prostatitis and diverticulitis GPs ranked the need for a better evidence base for antibiotic treatment and improved treatment guidelines as priority areas. At the time of the 2017 survey, there was no antibiotic prescribing guidance for these conditions in England; however, NICE have since launched their first antimicrobial prescribing guidance for both acute prostatitis (2018) [41] and diverticular disease (2019) [42]. There is still a need for a greater evidence base in both these conditions [41,42].

It is not surprising that suspected infection in the elderly was ranked as the top condition for which GPs required evidence to support their daily practice as this group has higher infectious disease morbidity [40,43]. The UK population is also getting older; the number of UK residents aged 65 and over has increased by 2.7 million in the past 25 years and is expected to rise by a further 8.6 million in the next 50 years [44]. This increase in life expectancy has a knock-on effect on our health services, with antibiotic prescribing rates being the highest in this age group [45]. In a recent study, GPs used antibiotic treatment both as a diagnostic aid and in an attempt to avoid hospital admission and felt that, in some cases, restrictions on antibiotic use potentially hampered optimal management of infection in this age group [46]. Similar to our findings, authors concluded that research that can fill the gaps in the evidence base is required in order to support GPs with their critical antimicrobial stewardship role in this population.

Over 60% of bacteraemia occurs in over 65 year olds who have a 13-fold higher risk of developing sepsis [47]. The need for a better evidence base for antibiotic treatment and improved treatment guidelines were ranked as the top two GP priorities.

## 4. Materials and Methods

An online questionnaire survey, based on a previous survey from 1999 [9], was used to collect data from GPs across the UK. For this study researchers chose to focus on 27 common conditions/illnesses based on their clinical expertise. The survey was designed and tested by researchers, GPs and microbiologists at Public Health England (PHE). The survey comprised three sections, with multiple fixed questions and one open question (Appendix A):

- Participant rating of 27 named illnesses/conditions based on how much more evidence they perceive is required to support daily practice.
- Participant selection of the top three illnesses/conditions that they perceive require further research, evidence and guidance, with participant identification of where those improvements are required.
- Demographic data collection.

The survey was implemented using SelectSurvey (SelectSurvey.NETv4, ClassApps LLC, Kansas City, MO, USA).

### 4.1. Survey Dissemination

A link to the survey and a covering letter were disseminated to GPs between June and November 2017 via the Royal College of General Practitioners newsletter (RCGP) (*n* ≥ 2000 individual members and to 230 practices); the regional Clinical Research Network (CRN) leads (*n* = 15), via email, for distribution to their members; and all Clinical Commissioning Group (CCG) medicine managers (*n* = 161) via e-mail, for distribution to their GPs.

### 4.2. Data Management

Data were exported from SelectSurvey to Stata (Stata Statistical Software: Release 13. StataCorp LP, College Station, TX, USA) for analysis. Survey items that asked respondents to rate priorities on a Likert scale were given an overall percentage score, which was calculated by dividing a weighted sum of individual responses (coded as very unimportant = 1, very important = 5) by the theoretical maximum score.

For the top three illness/condition sections of the survey where different respondents may have rated the same illness/condition in either the number 1, 2 or 3 position, the total number of respondents selecting a particular illness/condition were added together to give the final overall ranking.

### 4.3. Representativeness of the GP Sample

Statistical comparisons, using the Chi-square test (significant at the 5% level), were made between the survey respondents and all recognised GPs in the sample frame during the study period (Table 1). The GP characteristics data for the respondents were taken from their survey responses, while the data for all GPs in the sample frame were obtained from NHS Digital [11].

### 4.4. Comparison of Importance of Research between GPs from Research and Non-Research Practices

Research practices are defined as GP practices that actively take part in research projects. For each condition ordinal logistic regression was used to assess the association between research practice and rating, without adjusting for any other covariate. This association was also assessed after simultaneously adjusting for gender, years, location, rurality and audit, the remaining being omitted due to strong collinearity between them and the other covariates. Where this was not possible, a model was developed by means of a forwards stepwise approach wherein non-significant (at the 5% level), not substantially confounding covariates (a covariate was a substantial confounder if its removal resulted in a greater than 10% change in the odds ratios (ORs) of one or more of the parameters still in the model) were removed, but always retaining research practice. If none of the covariates were found to be significant or confounding, the unadjusted association between research practice and rating is presented. The proportional odds assumption was tested by means of a likelihood ratio test (LRT) and, if significant at the 5% level, a generalised ordered logit model was fitted wherein the proportionality assumption was relaxed for those parameters not meeting the criterion, as detailed in the reference. The likelihood ratio test LRT was used to obtain determine significance, except when the generalised ordered logit model was used, in which case the p-value was obtained from the Wald test. The measure of association for research practice quoted was the odds ratio (OR), together with 95% confidence intervals (CIs).

## 5. Conclusions

This survey has highlighted areas of topic prioritisation for the development of guidance and future research areas. Since the 1999 survey, investment in research, evidence-based treatment guidelines, training, clinical prediction tools and screening programmes for many of the common infections may have led to the decreased prioritisation of acute RTIs by GPs. The focus for research to support diagnostic and management guidance now needs to be on less common and chronic infections. We are encouraged that NICE and PHE have already developed antibiotic prescribing guidance for some of these conditions [31,35,37,41,42]; however, three of the top 10 conditions where GPs required evidence to support their daily practice future were for chronic or recurring conditions, i.e., chronic sinusitis, chronic cough, recurring UTI, for which there is currently little or no diagnostic, management or treatment guidelines.

## Figures and Tables

**Table 1:** Respondent reported age, sex and number of years in practice compared to national data, where available. Statistical comparisons made using the Chi-square test (significant at the 5% level).

Variable	Survey (a)(b)		England (c)(d) [11] *	
	Number	Percent of GPs	Number	Percent of GPs
**Age** (*p* = 0.260)	*n* = 367		*n* = 44,047	
20–30	17	5%	2110	5%
31–40	106	29%	10,363	31%
41–50	103	28%	9629	28%
51–60	110	30%	8677	24%
60+	31	8%	2677	8%
Unknown		-	1485	3%
**Sex** (*p* = 0.183)	*n* = 363		*n* = 43,966	
Male	150	41%	19,213	44%
Female	213	59%	23,659	54%
Unknown			1094	2%
**Research Practice** (*p* = 0.019)	*n* = 361		*n* = 7840	
Yes	174	48%	3293	42%
No	187	52%	4547	58%
**Years in practice**	*n* = 362			
0–5	75	21%	-	-
6–10	59	16%	-	-
11–15	59	16%	-	-
16–20	45	12%	-	-
20+	124	34%	-	-
**Region** * (*p* =< 0.05)	*n* = 288		*n* = 44,737	
North East	13	4%	2159	5%
North West	35	12%	5878	13%
Yorkshire and Humber	14	5%	4364	10%
East Midlands	22	8%	3490	8%
West Midlands	36	12%	4634	10%
East of England	11	4%	4463	10%
London	28	10%	7175	16%
South East	55	19%	7417	17%
South West	74	26%	4354	10%
Unknown			803	2%
**Locality**	*n* = 360			
urban	166	46%	-	-
suburban	140	39%	-	-
rural	54	15%	-	-

(a) Missing data removed. (b) Survey data completed by each respondent. (c) Reference data from NHS digital General Practice Workforce Final 31 December 2018, Experimental Statistics GP Tables Final—December 2018 (https://files.digital.nhs.uk/C4/7AD1A0/GPWDec18GP_v3.xlsx) (d) Research Practice Reference research data from the National Institute for Health research (NIHR) report in 2017. * There was no directly comparable data by region; therefore, reference data is by Health Education England (HEE) regions with Wessex being included in the South East.

**Table 2:** All 27 named illnesses/conditions in order of importance for further evidence to support daily practice.

Order of Importance for Evidence to Support Daily Practice			Condition/Illness	Top 3 Ranked by the Need for More Research, Evidence and Guidance	
Number of Respondents	Rank	Rank (1999^8^)		Rank	Number of Respondents
415	1	-	Suspected infection in the elderly	1	115
417	2	18	UTI (recurrent)	2	107
413	3	3	Surveillance of AMR in the community	1	115
418	4	5	Leg ulcers	5	70
413	5	17	Cough (persistent)	3	94
414	6	28	Cellulitis	4	89
404	7	6	Sinusitis (persistent)	9	28
412	8	-	Prostatitis	7	36
408	9	-	Diverticulitis	8	30
412	10	18	UTIs	10	25
409	11	17	Cough (acute)	6	47
406	12	4	Vaginal discharge	8	18
409	13	1	Lyme disease	9	28
411	14	-	Epididymitis/Orchitis	13	20
411	15	7	Otitis externa	12	22
413	16	11	Infection in returning travelers	11	23
410	17	6	Sinusitis (acute)	17	13
414	18	24	Hepatitis viral	14	19
411	19	19	Tuberculosis	18	8
406	20	14	HIV/AIDS	15	16
418	21	7	Otitis media	16	15
413	22	-	Insect Bites, possible skin infections	16	15
408	23	1	Chlamydia genital	20	4
415	24	26	Fungal nail infections in the community	22	23
415	25	-	Mastitis	19	7
415	26	-	AMR in returning travelers	10	25
414	27	10	Tonsillitis/Pharyngitis	19	7

**Table 3:** Evidence, research and guidance needs, ranked in order of importance, for each named condition. Conditions are listed in order of importance for further research, as outlined in Table 2.

Condition	Need for Better Evidence Base for Antibiotic Treatment in Primary Care		Need for Better Evidence Base for Self-Care and Non-Antibiotic Treatment in Primary Care		Need for Better Near Patient Antibiotic Resistance Test in Primary Care		Need for Improved Treatment Guidelines for Primary Care Staff		Need for Better Clinical Scores to Help Inform Management in Primary Care		Need For Better Point of Care Prognostic Tests In Primary Care	
	Rank	Mean	Rank	Mean	Rank	Mean	Rank	Mean	Rank	Mean	Rank	Mean
Suspected infection in the elderly	1	4.58	4	4.25	5	4.16	2	4.46	6	4.15	3	4.38
UTI (recurrent)	2	4.50	1	4.53	5	4.15	3	4.37	6	3.94	4	4.16
Surveillance of AMR in the community	1	4.64	2	4.60	4	4.25	5	4.21	6	3.98	3	4.27
Leg Ulcers	2	4.46	3	4.43	5	3.92	1	4.52	4	4.03	6	3.91
Cough (persistent)	3	4.31	1	4.43	6	3.76	2	4.42	5	4.02	4	4.05
Cellulitis	1	4.61	3	4.28	6	3.92	2	4.37	4	4.09	5	4.03
Sinusitis (persistent)	3	4.17	1	4.54	6	3.33	2	4.42	4	3.87	5	3.52
Prostatitis	1	4.65	3	3.94	5	3.53	2	4.38	3	3.94	4	3.74
Diverticulitis	1	4.48	3	4.40	6	3.33	2	4.41	4	4.07	5	3.70
UTIs	2	4.54	1	4.67	4	4.33	3	4.39	5	4.08	6	4.00
Cough (acute)	2	4.44	1	4.57	6	3.90	4	4.24	5	4.02	3	4.26
Vaginal Discharge	2	4.57	3	4.17	4	3.86	1	4.71	6	3.57	5	3.71
Lyme Disease	2	4.50	5	3.71	6	3.08	1	4.54	4	3.85	3	3.92
Epididymitis/Orchitis	1	4.42	3	4.05	5	3.74	2	4.37	4	3.88	6	3.68
Otitis Externa	2	4.20	2	4.20	5	3.25	1	4.42	3	3.95	4	3.60
Infection In Returning Travellers	1	4.57	3	4.20	6	4.00	2	4.48	5	4.05	4	4.19
Sinusitis (acute)	2	4.50	1	4.62	5	3.58	3	4.00	4	3.85	3	4.00
Hepatitis (viral)	5	3.07	3	3.64	6	2.53	1	4.21	2	4.20	3	3.67
Tuberculosis	1	4.43	5	3.57	4	3.86	1	4.43	2	4.14	3	4.00
HIV/AIDS	4	3.93	2	4.14	5	3.57	1	4.36	4	3.93	3	4.00
Otitis Media	2	4.58	1	4.67	6	3.92	3	4.50	4	4.25	5	4.00
Insect Bites, possible skin infections	2	4.29	1	4.50	5	3.57	3	3.93	4	3.79	3	3.93
Chlamydia (genital)	2	4.00	4	2.33	1	4.33	3	3.00	4	2.33	2	4.00
Fungal nail infections in the community	3	3.84	1	4.47	5	3.16	2	4.37	4	3.32	4	3.32
Mastitis	2	4.20	3	3.75	6	2.80	1	4.40	4	3.67	5	3.40
AMR in returning travellers	2	4.36	3	4.25	3	4.25	1	4.42	4	4.09	5	4.04
Tonsillitis/Pharyngitis	2	4.50	1	4.67	5	3.33	3	4.17	3	4.17	4	3.83
Overall scoring	1	4.35	3	4.21	5	3.68	2	4.31	4	3.90	4	3.90

## Appendix A. GP Questionnaire Survey

### Section 1

1. On a scale of 1–5, please indicate how much more evidence you would like to see for each of the following conditions to support your daily clinical practice.
  (Tick one box for each statement. Please note that conditions are in alphabetical order)
  AMR in returning travellers
    Cellulitis
    Chlamydia Genital
    Cough Acute
    Cough Persistent
    Diverticulitis
    Epididymitis/Orchitis
    Fungal nail infections in the community
    Hepatitis Viral
    HIV/AIDS
    Infection in Returning Travellers
    Insect Bites, possible skin infections
    Leg Ulcers
    Lyme Disease
    Mastitis
    Otitis Externa
    Otitis Media
    Prostatitis
    Sinusitis Acute
    Sinusitis Persistent
    Surveillance of AMR in the community
    Suspected Infection in The Elderly
    Tonsillitis/Pharyngitis
    Tuberculosis
    UTI Recurrent
    UTIs
    Vaginal Discharge
2. Please specify up to three conditions/illnesses not mentioned that you think require further research
  On a scale of 1–5, please indicate how much more evidence you would like to see for your chosen illness/condition to support your daily clinical practice. (Tick one box for each statement)
  Optional extra 1
    Optional extra 2
    Optional extra 3

### Section 2

1. From the list of illnesses/conditions you just rated, which top 3 illnesses/conditions do you feel require further research, evidence and guidance?
  - Illness condition 1
  - Illness condition 2
  - Illness condition
2. In relation to this illness/condition (respondents will only be shown their 3 selected illness/conditions), please indicate how important you feel improvements in each of the following areas are?
  - Need for better evidence base for antibiotic treatment in primary care
  - Need for better evidence base for self-care and non-antibiotic treatment in primary care
  - Need for better near patient antibiotic resistance test in primary care
  - Need for improved treatment guidelines for primary care staff
  - Need for better clinical scores to help inform management in primary care
  - Need for better point of care prognostic tests in primary care

### Section 3

1. How did you receive this questionnaire?
  - Royal College of General Practitioners (RCGP)
  - NIHR Clinical Research Network ((CRN) (Primary Care))
  - Clinical Commissioning Group (CCG)
  - Colleague
  - Other, please specify
2. Age (years)
  - 20–30
  - 31–40
  - 41–50
  - 51–60
  - 60+
3. Sex
  - Male
  - Female
4. How many years have you been a practicing GP?
  - 0–5
  - 6 to 10
  - 11 to 15
  - 16–20
  - 20+
5. Did you carry out an antibiotic/infection audit in the past 12 months?
  - Yes
  - No
6. Where are you a general practitioner?
  - England
  - Scotland
  - Wales
  - Northern Ireland
7. Which region is your practice in?
  Please select one
  - North East
  - North West
  - Yorkshire and Humber
  - East Midlands
  - West Midlands
  - East of England
  - London
  - South East
  - South West
8. How would you describe your practice geographical location?
  - rural
  - urban
  - suburban
9. Is your practice a research practice?
  - Yes
  - No

## Appendix B

**Table A1:** Comparison between researchers and non-researchers in Question 1 responses. The *p*-value obtained from the likelihood ratio test (LRT), except where indicated otherwise.

Condition	Responses	Non-Research	Research	Unadjusted			Adjusted		
	Further Research is	(*n*)	(*n*)						
AMR in returning travellers	Very Unimportant	20	22	OR 0.7	[95% CI 0.48–1.01];	*p* = 0.06	OR 0.76	[95% CI 0.50–1.14];	*p* = 0.18
	Unimportant	44	50						
	Neutral	55	48						
	Important	39	32						
	Very important	26	14						
Cellulitis	Very Unimportant	6	10	OR 0.99	[95% CI 0.68–1.46];	*p* = 0.98	OR 0.96	[95% CI 0.63–1.45];	*p* = 0.8
	Unimportant	19	16						
	Neutral	42	34						
	Important	70	72						
	Very important	42	38						
Chlamydia genital	Very Unimportant	16	17	OR 0.89	[95% CI 0.61–1.31];	*p* = 0.6	OR 0.92	[95% CI 0.61–1.40];	*p* = 0.7
	Unimportant	40	45						
	Neutral	75	58						
	Important	40	34						
	Very important	9	12						
Cough (acute)	Very Unimportant	19	17	OR 1.2	[95% CI 0.82–1.74];	*p* = 0.3	OR 1.29	[95% CI 0.88–1.90];	*p* = 0.19
	Unimportant	36	30						
	Neutral	47	43						
	Important	49	44						
	Very important	28	35						
Cough (persistent)	Very Unimportant	8	6	OR 1.2	[95% CI 0.82–1.76];	*p* = 0.3	OR 1.31	[95% CI 0.89–1.95];	*p* = 0.17 ^*^
	Unimportant	15	11						
	Neutral	42	35						
	Important	70	69						
	Very important	47	48						
Diverticulitis	Very Unimportant	11	6	OR 1.11	[95% CI 0.76–1.63];	*p* = 0.6	OR 1.29	[95% CI 0.85–1.95];	*p* = 0.2 *
	Unimportant	23	29						
	Neutral	58	45						
	Important	63	62						
	Very important	23	25						
Epididymitis	Very Unimportant	14	9	OR 1.03	[95% CI 0.70–1.51];	*p* = 0.9	1	[95% CI 0.66–1.51];	*p* = 0.99
	Unimportant	28	34						
	Neutral	70	63						
	Important	54	44						
	Very important	13	19						
Community fungal nail infection	Very Unimportant	38	29	OR 0.97	[95% CI 0.67–1.40];	*p* = 0.9	OR 1.19	[95% CI 0.80–1.78];	*p* = 0.4
	Unimportant	32	43						
	Neutral	40	37						
	Important	46	40						
	Very important	25	23						
Hepatitis viral	Very Unimportant	16	11	OR 0.98	[95% CI 0.67–1.43];	*p* = 0.9	OR 0.98^x^	[95% CI 0.67–1.43^x^];	*p* = 0.9 ^x^
	Unimportant	38	42						
	Neutral	66	62						
	Important	45	31						
	Very important	17	22						
HIV/AIDS	Very Unimportant	15	16	OR 0.9	[95% CI 0.61–1.31];	*p* = 0.6	OR 0.90^x^	[95% CI 0.61–1.31^x^];	*p* = 0.6 ^x^
	Unimportant	43	44						
	Neutral	64	56						
	Important	38	23						
	Very important	20	25						
Infection in returning travellers	Very Unimportant	13	11	OR 0.78	[95% CI 0.53–1.13];	*p* = 0.19	OR 0.83	[95% CI 0.55–1.25];	*p* = 0.4
	Unimportant	34	44						
	Neutral	60	56						
	Important	50	42						
	Very important	23	17						
Insect bites	Very Unimportant	19	21	OR 0.98	[95% CI 0.68–1.43];	*p* = 0.9	OR 1.17	[95% CI 0.78–1.76];	*p* = 0.5 *
	Unimportant	43	44						
	Neutral	63	46						
	Important	43	45						
	Very important	12	13						
Leg ulcers	Very Unimportant	5	7	OR 1.12	[95% CI 0.77–1.64];	*p* = 0.5	OR 1.11	[95% CI 0.73–1.67];	*p* = 0.6 *
	Unimportant	15	15						
	Neutral	38	35						
	Important	76	56						
	Very important	48	58						
Lyme disease	Very Unimportant	16	13	OR 0.91	[95% CI 0.62–1.32];	*p* = 0.6	OR 1.03	[95% CI 0.68–1.55];	*p* = 0.9
	Unimportant	32	44						
	Neutral	62	50						
	Important	43	38						
	Very important	24	25						
Mastitis	Very Unimportant	19	21	OR 0.91	[95% CI 0.62–1.32];	*p* = 0.6	OR 0.91^x^	[95% CI 0.62–1.32^x^];	*p* = 0.6 ^x^
	Unimportant	40	41						
	Neutral	74	60						
	Important	38	38						
	Very important	11	9						
Otitis external	Very Unimportant	17	21	OR 0.91	[95% CI 0.64–1.36];	*p* = 0.7	OR 0.99	[95% CI 0.67–1.47];	*p* = 0.97 *
	Unimportant	37	36						
	Neutral	51	40						
	Important	55	45						
	Very important	22	25						
Otitis media	Very Unimportant	19	20	OR 0.96	[95% CI 0.66–1.39];	*p* = 0.8	OR 1.06	[95% CI 0.70–1.59];	*p* = 0.8 *
	Unimportant	46	41						
	Neutral	51	46						
	Important	46	48						
	Very important	22	16						
Prostatitis	Very Unimportant	11	3	OR 1.6	[95% CI 1.09–2.34];	*p* = 0.02	OR 1.53	1.02–2.32];	*p* = 0.04 *
	Unimportant	21	22						
	Neutral	77	54						
	Important	52	60						
	Very important	21	30						
Sinusitis (acute)	Very Unimportant	22	19	OR 0.99	[95% CI 0.68–1.44];	*p* = 0.9	OR 1.12	[95% CI 0.75–1.69];	*p* = 0.6
	Unimportant	39	38						
	Neutral	49	45						
	Important	43	49						
	Very important	26	18						
Sinusitis (persistent)	Very Unimportant	9	8	OR 0.96	[95% CI 0.66–1.42];	*p* = 0.8	OR 1.19	[95% CI 0.78–1.82];	*p* = 0.4
	Unimportant	20	17						
	Neutral	43	45						
	Important	75	67						
	Very important	31	28						
Surveillance of AMR in community	Very Unimportant	5	7	OR 1.16	[95% CI 0.79–1.72];	*p* = 0.4	OR 1.31	[95% CI 0.86–2.02];	*p* = 0.2
	Unimportant	15	12						
	Neutral	22	19						
	Important	58	46						
	Very important	81	85						
Suspected infection in elderly	Very Unimportant	6	1	OR 1.19	[95% CI 0.81–1.76];	*p* = 0.4	OR 1.4	[95% CI 0.91–2.16];	*p* = 0.13 *
	Unimportant	8	7						
	Neutral	23	19						
	Important	74	71						
	Very important	72	71						
Tonsillitis	Very Unimportant	25	30	OR 0.89	[95% CI 0.61–1.29];	*p* = 0.5	OR 0.94	[95% CI 0.62–1.41];	*p* = 0.8
	Unimportant	49	44						
	Neutral	52	48						
	Important	43	34						
	Very important	12	14						
Tuberculosis	Very Unimportant	18	19	OR 0.82	[95% CI 0.56–1.19];	*p* = 0.3	0.85	[95% CI 0.57–1.29];	*p* = 0.5
	Unimportant	36	41						
	Neutral	58	56						
	Important	54	31						
	Very important	15	21						
UTI (recurrent)	Very Unimportant	4	5	1.22	[95% CI 0.83–1.79];	*p* = 0.3	1.38	[95% CI 0.91–2.12];	*p* = 0.13 *
	Unimportant	6	6						
	Neutral	34	23						
	Important	74	70						
	Very important	64	68						
UTIs	Very Unimportant	13	12	1.05	[95% CI 0.72–1.54];	*p* = 0.8	1.2	[95% CI 0.79–1.80];	*p* = 0.4
	Unimportant	33	28						
	Neutral	53	55						
	Important	58	38						
	Very important	26	34						
Vaginal discharge	Very Unimportant	10	10	1	[95% CI 0.68–1.46];	*p* = 0.98	1.19	[95% CI 0.78–1.80];	*p* = 0.4 *
	Unimportant	32	32						
	Neutral	64	61						
	Important	55	43						
	Very important	15	21						

* Wald *p*-value ^x^ Unadjusted.

## References

[1] Baird B., Charles A., Honeyman M., Maguire D., Das P. (2016). Understanding Pressures in General Practice.

[2] NHS Digital (2018). GP Appointments. Comparison to Other Collections.

[3] Public Health England (2019). English Surveillance Programme for Antimicrobial Utilisation and Resistance (ESPAUR) Report 2018–2019.

[4] Nugent C., Patterson L., Sartaj M. Surveillance of Antimicrobial Use and Resistance in Northern Ireland, Annual Report 2018.

[5] Public Health Wales. Antibacterial Usage in Primary Care in Wales 2013/14–2017/18.

[6] Public Health England (2018). Research Reveals Levels of Inappropriate Prescriptions in England.

[7] Pouwels K.B., Dolk F.C.K., Smith D.R.M., Robotham J.V., Smieszek T. (2018). Actual versus ‘ideal’ antibiotic prescribing for common conditions in English primary care. J. Antimicrob. Chemother..

[8] Germeni E., Frost J., Garside R., Rogers M., Valderas J.M., Britten N. (2018). Antibiotic prescribing for acute respiratory tract infections in primary care: An updated and expanded meta-ethnography. Br. J. Gen. Pract..

[9] McNulty C.A., Smith G.E., Graham C. (2001). PHLS primary care consultation--infectious disease and primary care research and service development priorities. Commun. Dis. Public Health.

[10] McNulty C.A., Thomas M.D. (2002). PHLS laboratory services and primary care needs. Prescriber.

[11] NHS Digital (2019). General Practice Workforce, Final 30 September 2018, Experimental Statistics.

[12] Fryrear A. What’s a Good Response Rate?. https://www.surveygizmo.com/resources/blog/survey-response-rates/.

[13] Porter B. Tips and Tricks to Improve Survey Response Rate. https://www.surveymonkey.com/curiosity/improve-survey-response-rate/.

[14] NHS Digital (2018). General Practice Trends in the UK to 2017.

[15] McNulty C.A.M., Freeman E., Bowen J., Shefras J., Fenton K.A. (2004). Diagnosis of genital chlamydia in primary care: An explanation of reasons for variation in chlamydia testing. Sex. Transm. Infect..

[16] Radcliffe K., Jusuf I., Cowan F., Fitzgerald M., Wilson J. (1999). UK national guidelines on sexually transmitted infections and closely related conditions. Sex. Transm. Infect..

[17] British Association for Sexual Health and HIV (BASHH) BASHH Guidelines. https://www.bashh.org/guidelines.

[18] National Chlamydia Screening Programme (NCSP) Information, Data, Guidance and Resources about the NCSP. https://www.gov.uk/government/collections/national-chlamydia-screening-programme-ncsp.

[19] Lewis J., White P.J. (2018). Changes in chlamydia prevalence and duration of infection estimated from testing and diagnosis rates in England: A model-based analysis using surveillance data, 2000-15. Lancet Public Health.

[20] Finch R.G., Low D.E. (2002). A critical assessment of published guidelines and other decision-support systems for the antibiotic treatment of community-acquired respiratory tract infections. Clin. Microbiol. Infect..

[21] Spurling G.K.P., Del Mar C.B., Dooley L., Foxlee R., Farley R. (2017). Delayed antibiotic prescriptions for respiratory infections. Cochrane Database Syst. Rev..

[22] Hay A.D., Redmond N.M., Turnbull S., Christensen H., Thornton H., Little P., Thompson M., Delaney B., Lovering A.M., Muir P. (2016). Development and internal validation of a clinical rule to improve antibiotic use in children presenting to primary care with acute respiratory tract infection and cough: A prognostic cohort study. Lancet Respir. Med..

[23] Centor R.M., Witherspoon J.M., Dalton H.P., Brody C.E., Link K. (1981). The diagnosis of strep throat in adults in the emergency room. Med. Decis. Mak. Int. J. Soc. Med. Decis. Mak..

[24] Little P., Hobbs F.D., Moore M., Mant D., Williamson I., McNulty C., Cheng Y.E., Leydon G., McManus R., Kelly J. (2013). Clinical score and rapid antigen detection test to guide antibiotic use for sore throats: Randomised controlled trial of PRISM (primary care streptococcal management). BMJ.

[25] Guerra B., Gaveikaite V., Bianchi C., Puhan M.A. (2017). Prediction models for exacerbations in patients with COPD. Eur. Respir. Rev..

[26] Lundberg T., Hellstrom S., Sandstrom H. (2013). Development and validation of a new grading scale for otitis media. Pediatric Infect. Dis. J..

[27] Jones L.F., Hawking M.K.D., Owens R., Lecky D., Francis N.A., Butler C., Gal M., McNulty C.A.M. (2017). An evaluation of the TARGET (Treat Antibiotics Responsibly; Guidance, Education, Tools) Antibiotics Toolkit to improve antimicrobial stewardship in primary care—Is it fit for purpose?. Fam. Pract..

[28] Finch R.G., Metlay J.P., Davey P.G., Baker L.J. (2004). Educational interventions to improve antibiotic use in the community: Report from the International Forum on Antibiotic Resistance (IFAR) colloquium, 2002. Lancet Infect. Dis..

[29] Tan M.K.H., Luo R., Onida S., Maccatrozzo S., Davies A.H. (2019). Venous Leg Ulcer Clinical Practice Guidelines: What is AGREEd?. Eur. J. Vasc. Endovasc. Surg..

[30] Public Health England Guidance: Managing Common Infections: Guidance for Primary Care. https://www.gov.uk/government/publications/managing-common-infections-guidance-for-primary-care.

[31] National Institute for Health and Care Excellence Leg Ulcer Infection: Antimicrobial Prescribing. NICE Guideline [NG152]. https://www.nice.org.uk/guidance/ng152.

[32] Fokkens W.J., Lund V.J., Mullol J., Bachert C., Alobid I., Baroody F., Cohen N., Cervin A., Douglas R., Gevaert P. (2012). EPOS 2012: European position paper on rhinosinusitis and nasal polyps 2012. A summary for otorhinolaryngologists. Rhinology.

[33] Head K., Chong L.Y., Piromchai P., Hopkins C., Philpott C., Schilder A.G.M., Burton M.J. (2016). Systemic and topical antibiotics for chronic rhinosinusitis. Cochrane Database Syst. Rev..

[34] Rudmik L., Soler Z.M. (2015). Medical Therapies for Adult Chronic Sinusitis: A Systematic Review. JAMA.

[35] National Institute for Health and Care Excellence Sinusitis (Acute): Antimicrobial Prescribing NICE Guideline [NG79]. https://www.nice.org.uk/guidance/ng79.

[36] NHS England Reducing Gram Negative Blood Stream Infections (BSI) across the Whole Health Economy. https://www.england.nhs.uk/publication/part-a-reducing-gram-negative-blood-stream-infections-bsi-across-the-whole-health-economy/.

[37] National Institute for Health and Care Excellence Antimicrobial Prescribing Guidelines. https://www.nice.org.uk/about/what-we-do/our-programmes/nice-guidance/antimicrobial-prescribing-guidelines.

[38] HM Government Tackling Antimicrobial Resistance 2019–2024. The UK’s Five-Year National Action Plan. https://assets.publishing.service.gov.uk/government/uploads/system/uploads/attachment_data/file/784894/UK_AMR_5_year_national_action_plan.pdf.

[39] HM Government Contained and Controlled. The UK’s 20-Year Vision for Antimicrobial Resistance. https://assets.publishing.service.gov.uk/government/uploads/system/uploads/attachment_data/file/773065/uk-20-year-vision-for-antimicrobial-resistance.pdf.

[40] Public Health England Tuberculosis in England: 2018 Presenting Data to End of 2017. https://assets.publishing.service.gov.uk/government/uploads/system/uploads/attachment_data/file/774091/TB_Annual_Report_2018_2.pdf.

[41] National Institute for Health and Care Excellence Prostatitis (Acute): Antimicrobial Prescribing NICE Guideline [NG110]. https://www.nice.org.uk/guidance/ng110/history.

[42] National Institute for Health and Care Excellence Diverticular Disease: Antimicrobial Prescribing.

[43] Kline K.A., Bowdish D.M. (2016). Infection in an aging population. Curr. Opin. Microbiol..

[44] Storey A., Office for National Statistics (2018). Living Longer: How Our Population Is Changing and Why It Matters.

[45] Dolk F., Pouwels K., Smith D., Robotham J., Smieszek T. (2018). Antibiotics in primary care in England: Which antibiotics are prescribed and for which conditions?. J. Antimicrob. Chemother..

[46] Hayward G.N., Moore A., McKelvie S., Lasserson D.S., Croxson C. (2019). Antibiotic prescribing for the older adult: Beliefs and practices in primary care. J. Antimicrob. Chemother..

[47] Poolman J.T., Anderson A.S. (2018). Escherichia coli and Staphylococcus aureus: Leading bacterial pathogens of healthcare associated infections and bacteremia in older-age populations. Expert Rev. Vaccines.

